# Costas DFC-Based Random Stepped Wideband Waveform for Interference Countermeasure in SAR Imagery

**DOI:** 10.3390/s22093197

**Published:** 2022-04-21

**Authors:** GanE Dai, Lei Zhang, Sha Huan

**Affiliations:** 1 School of Electronics and Communication Engineering, Sun Yat-sen University, Shenzhen 518107, China; daige3@mail2.sysu.edu.cn; 2 School of Electronics and Communication Engineering, Guangzhou University, Guangzhou 510006, China; speeshuan@gzhu.edu.cn

**Keywords:** anti-jamming, synthetic aperture radar (SAR), Costas, dicrete frequency code (DFC)

## Abstract

Interference in SAR imagery will induce false targets or form a mask in specific areas to prevent accurate scene assessment. Traditional anti-jamming methods based on waveform agility require a trade-off between anti-jamming performance and imaging quality in waveform design. In this paper, we proposed a SAR ECCM scheme including a Costas DFC-based random stepped wideband waveform and corresponding imaging processing method. The waveform exhibits high flexibility against forwarding interference due to the decomposition of a wideband signal into multiple pulses with different Costas discrete frequency encoding, carrier frequency and phase modulation. Furthermore, the combination of FCDC and the imaging processing successfully overcomes the Doppler sensitivity of the proposed waveform. Extensive simulations confirmed the superiority of this waveform and processing method under different interference strategies.

## 1. Introduction

Synthetic aperture radar (SAR) has a capability of high-resolution imaging under a variety of weather and illumination conditions, which is of great value in both civil and military fields [1]. With the development of SAR technology, multiple specific electronic countermeasures (ECM) have been developed in past decades to prevent accurate battle scene assessment. Jamming techniques for SAR are mainly divided into incoherent jamming [2] and coherent jamming [3]. Since SAR achieves high processing gain utilizing the coherent characteristics of intra- and interpulses, the efficiency of the incoherent jamming technique such as the barrage jamming may be significantly depressed. Thus, as a coherent jamming technology, deception jamming has been proved to be indispensable for effectively counteracting SAR [4].

A deception jammer intercepts the victim radar pulses, employs digital radio frequency memory (DRFM) to modify these pulses to the best of the jammer’s capabilities rapidly and accurately, and retransmits them back to the victim radar. The jamming signal obtains a certain processing gain from pulse compression or coherent processing, inducing modulated false targets to confuse the information acquisition with relatively low transmitting power. However, due to the processing delay, the direct–re-transimitted signal will arrive at the receiver later than the true echo. To prevent false targets that can only be generated with positive range offsets, some jamming strategies have been proposed. Interrupted sampling repeater jamming (ISRJ) can form several deception targets spreading along the range direction, and some false targets can be designed to locate ahead of the real echo position [5,6,7]. Frequency shift jamming utilizes the time-frequency coupling characteristics of traditional SAR waveforms to generate point-like false targets or area coverage effects through different frequency shift strategies in range and azimuth [8,9]. It can also produce false targets with negative range offset, and even achieve active echo cancellation through amplitude and phase matching.

Meanwhile, many electronic counter-countermeasure (ECCM) technologies have been developed to suppress the deception jamming effect on SAR. Excavating and enriching the degrees of freedom for the radar signal is an attractive and effective way. Multichannel SAR [10] and multistatic SAR [11] provide more spatial degrees of freedom than conventional single-channel SAR. The jammer has difficulty processing multiple signals simultaneously, which introduces differences in multi-channel signal characteristics between the interference signal and the real echo, so the jamming signal can be easily identified and suppressed [12,13]. However, the relatively high cost of the multi-channel SAR limits its practical application. Waveform diversity increases the degrees of freedom in the time domain. It only requires good cross-correlation characteristics between multiple waveforms. Under the assumption that the jamming signal does not return in the current pulse repetition interval (PRI), good cross-correlation means that the jamming signal and subsequent echoes are orthogonal. So the coherence of the jamming signal is destroyed, resulting in jamming suppression. Phase-perturbed LFM is proposed with partial random perturbation on the signal phase distribution [14,15]. The waveform ECCM performance can also be improved by performing random recombination after the LFM segmentation [16]. Random phase modulation and chirp rate perturbation on linear frequency modulation (LFM) waveforms were developed for ECCM SAR [17,18]. This waveform addressed the Doppler-induced performance degradation caused by most other diversity schemes. Ref. [19] shows the advantages of OFDM-coded radar signals with random sub-band composition in deception jamming scenarios. OFDM can achieve better orthogonality than LFM perturbation, but requires special handling to the high peak-to-average power ratio (PAPR). Ref. [20] preliminarily verified the feasibility of Costas as a low probability of intercept (LPI) waveform for a notional SAR platform. However, all the inter-pulse diversity is invalid when subjected to ISRJ, because for the waveform with the large time-bandwidth product adopted by SAR for high gain; ISRJ jammer typically works within the current pulse period [6]. Ref. [16] improves the anti-ISRJ and imaging performance by the joint phase-coded waveform and mismatch filter design. However, this method requires the jamming parameters as a priori information and is sensitive to the duty cycle and period of ISRJ. Some research transformed the ECCM problem into a sparse optimization problem of extracting the time-frequency features of the jamming signal within a dynamic synthetic aperture [21,22], but the performance of the algorithm in dense target and low SNR scenarios needs to be further verified. The stepped frequency SAR can shorten the pulse width and time width of the waveform pulse, and has the ability to skip frequencies that might be corrupted from the jammer [23,24]. However, the existing stepped frequency SAR pulse waveform has a simple structure and is easily interfered with by the jammer after identifying the frequency hopping mode. So, there are still many challenges in waveform design and SAR signal processing methods to improve the anti-interference ability of SAR.

In this paper, we decompose this complex problem into two subproblems, waveform diversity design and an SAR processing method for Doppler-sensitive waveforms. We present a random stepped frequency synthesized wideband signal with different Costas discrete frequency coding in each pulse (RSF-DC-DFC). Random stepped frequency modulation is adopted to decompose the large time-bandwidth signal into several pulses with random frequency diversity, reducing the instantaneous bandwidth of the system. Pulses with smaller duration and bandwidth increases the difficulty of interception, and make it hard for the ISRJ jamming signal to act on the current pulse. Frequency diversity ensures sufficient orthogonality for all the pulses within the waveform which can resist inter-pulse forwarding interference. Costas discrete frequency coding (DFC) is applied in each pulse, combined with random stepping inter-pulse, so the synthetic wideband waveform overcomes the range-Doppler coupling of linear modulation. Therefore, this waveform can effectively counter the frequency-shift interference. To improve the sidelobe performance, each pulse within the waveform is modulated with a different Costas array. At the same time, a full cell Doppler correction (FCDC) is embedded in the coherent processing flow to deal with the Doppler sensitivity of the proposed RSF-DC-DFC. Corrections are performed separately for each Doppler grid in the Doppler domain before the frequency synthesis, which ensures the imaging quality of SAR. This SAR processing method poses no limit on the Costas coding choice, the random stepped frequency or the phase modulation between the pulses. Flexible modulation of RSF-DC-DFC can provide sufficient robustness against the jammer identification and improve the anti-jamming performance of SAR.

This paper is organized as follows. In Section 2, the RSF-DC-DFC signal model is constructed and the detection performance of the waveform is analyzed. Section 3 gives the details of the SAR ECCM strategy and the coherent SAR imaging process based on RSF-DC-DFC waveform. The simulation results are discussed in Section 4. Finally, the conclusion is drawn in Section 5.

## 2. Signal Modeling and Characteristics

### 2.1. RSF-DC-DFC Signal Construction

The RSF-DC-DFC signal is a two-dimensional frequency-coded waveform. It contains *N* transmission pulses modulated by a random frequency code. Each pulse is a discrete frequency-coding signal according to a specific Costas array. The baseband waveform of RSF-DC-DFC can be expressed as

(1) $$S\left(t\right)=\sum_{n=1}^{N}\mathrm{rect}\left(\frac{t-t_{p\_n}}{T_{p}}\right)P_{n}\left(t-t_{p\_n}\right)e^{j2\pi f_{p\_n}\left(t-t_{p\_n}\right)},$$

where

(2) $$P_{n}\left(t\right)=\sum_{m=1}^{M}\mathrm{rect}\left(\frac{t-t_{sp\_m}}{T_{sp}}\right)e^{j2\pi f_{mn}\left(t-t_{sp\_m}\right)}.$$

rect(·) is the rectangular function, $T_{r}$ is the pulse-repetition interval and $t_{p\_n}=\left(n-1\right)T_{r}$ describes the discrete slow time. *M* represents the order of the Costas array in each pulse, and each pulse of length $T_{p}$ is divided into *M* contiguous sub-pulses of equal duration $T_{sp}$. $t_{sp\_m}=\left(m-1\right)T_{sp}$ is the discrete time of each sub-pulse.

$f_{p\_n}$ is the center frequency of the *n*th pulse. It can be presented as

(3) $$f_{p\_n}=\zeta_{n}B_{p},\;n=1,2,\cdots ,N.$$

In (3), $\zeta_{n}$ is the *n*th number in a random arrangement of [1,N]. If *B* is defined as the RSF-DC-DFC bandwidth, $B_{p}=B/N$ is the bandwidth of each pulse.

**Figure 1:**
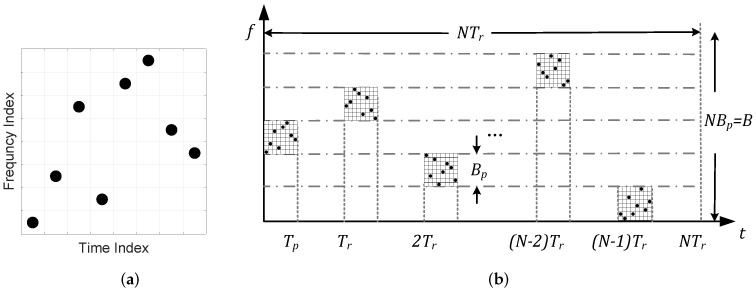
RSF-DC-DFC Wavefrom diagram: (**a**) a Costas array of order 8; (**b**) TF characteristics of the RSF-DC-DFC Wavefrom.

$f_{mn}$ is the frequency of each sub-pulse based on the Costas array, and the Costas array of each pulse is different in this waveform. The Costas array is a special permutation matrix. The permutation matrix is a *n*-order square matrix with exactly one 1 per row and colunm, and the remaining elements are 0. As shown in Figure 1a, the box with a dot indicates element 1, and the blank box represents element 0. The key feather of the Costas array is that all the vectors between any two dots are distinct. It means the new matrix generated by translation along the horizontal and vertical axis will produce only one coincidence at most with the original matrix [25]. When the rows and columns are applied as the indices of the sub-pulse time and frequency, respectively, this feather brings nearly ideal ambiguity function characteristics to the Costas-DFC signal. The signal after the match filter will have a high compression value with low sidelobes. RSF-DC-DFC modulates different Costas-DFC waveform with random frequency. The time-frequency (TF) characteristics of the RSF-DC-DFC signal is shown in Figure 1b.

**Figure 2:**
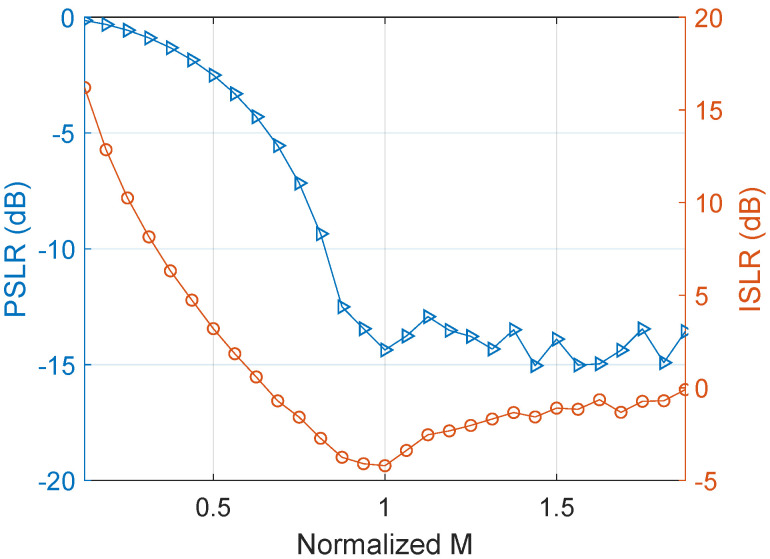
Comparison of the sidelobe performance of Costas DFC with different M under fixed pulse duration and bandwidth.

The order selection has to be made before the Costas array selection. The time-bandwidth product of the pulse is known as $T_{p}B_{p}$. Figure 2 exhibits the effect of the *M* value on the Costas DFC pulse compression performance under fixed pulse duration and bandwidth. Here, the peak sidelobe ratio (PSLR) and integrated sidelobe ratio (ISLR) is applied as the evaluation criteria, and *M* is normalized according to $\sqrt{T_{p}B_{p}}$. PSLR declines gradually when the normalized *M* increases, and becomes stable after reaching 1. ISLR reaches a minimum value when the normalized *M* value is 1.

Therefore, following the Nyquist choice [26], *M* can be calculated by the formula below.

(4) $$M=\mathrm{round}\left(\sqrt{T_{p}B_{p}}\right)\;$$

When *M* is below 26, a check matrix can be used to verify whether a permutation matrix results from exhaustive searches is a Costas array. For the orders above 27, the number-theoretic generators and extensions are commonly adopted to obtain the Costas array. Ref. [27] is a database available on the IEEE DataPort, which includes all known Costas arrays below an order of 1030 using the methodology in [28]. In this paper, *N* Costas arrays are randomly picked from this database as the frequency codes for each pulse, denoted by $C_{mn}$. When *M* and $C_{mn}$ are determined, $f_{mn}$ can be expressed as

(5) $$f_{mn}=C_{mn}B_{sp}\;m=1,2,\cdots ,M,n=1,2,\cdots ,N,$$

where $B_{sp}=\frac{B_{p}}{M}$ is the sub-pulse bandwidth.

### 2.2. The Ambiguity Function

Radar range and velocity profile reconstruction can be performed via the ambiguity function (AF). The definition of AF in the integral format is given as follows.

(6) $$\chi \left(\tau ,\xi \right)=\int_{-\infty }^{+\infty }S\left(t\right)S^{*}\left(t-\tau \right)e^{j2\pi \xi t}dt\;$$

The unambiguous range of Random Costas-DFC corresponds to $NT_{r}$. Due to the frequency orthogonality of the pulses, only the AF within the $T_{p}$ delay will be discussed in this paper.

**Figure 3:**
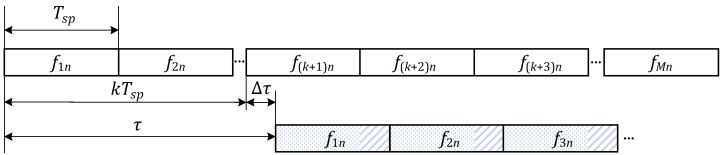
Frequency code relationship and sub-pulse division with delay τ.

In Figure 3, taken $T_{sp}$ as the time unit, τ can be represented by the integer part *k* and decimal part Δτ.

(7) $$\tau =kT_{sp}+\Delta \tau ,\;k=0,1,\cdots ,M-1$$

The existence of τ makes the sub-pulses modulated by $f_{mn}$ misaligned with the original signal. In the overlap interval of the two signals, each sub-pulse of the delayed signal is divided into a front part marked by a dot and an end part marked by a stripe, with the length of $T_{sp}-\Delta \tau$ and Δτ, respectively. The front part of the delayed *m*-th sub-pulse corresponds to the $f_{m+k}$ frequency code of the original signal, and the end part of this sub-pulse corresponds to the $f_{m+k+1}$ code. Therefore, the integral operation of AF can be divided into two parts according to the division of the sub-pulse.

(8) $$\chi \left(\tau ,\xi \right)=\sum_{n=1}^{N}\sum_{m=1}^{M-k}\chi_{sp\_f}\left(m,n,k,\Delta \tau ,\xi \right)+\sum_{n=1}^{N}\sum_{m=1}^{M-k-1}\chi_{sp\_e}\left(m,n,k,\Delta \tau ,\xi \right)\;$$

First, the front part of the *m*-th sub-pulse in the *n*-th pulse is discussed. Note that the *m* here refers to the sub-pulse number of the delayed conjugate signal. The position of this sub-pulse is simplified as $t_{mn}=t_{sp\_m}+t_{p\_n}$. The AF of this front part can be derived as:

(9) $$\begin{array}{cc} \; & \;\chi_{sp\_f}\left(m,n,k,\Delta \tau ,\xi \right)\\ = & \;\int_{-\frac{T_{sp}}{2}+t_{mn}+\tau }^{\frac{T_{sp}}{2}+t_{mn}+kT_{sp}}e^{j2\pi f_{mn}\left(t-t_{mn}\right)}e^{j2\pi f_{p\_n}\left(t-t_{p\_n}\right)}e^{-j2\pi f_{mn}\left(t-t_{mn}-\tau \right)}e^{-j2\pi f_{p\_n}\left(t-t_{p\_n}-\tau \right)}e^{j2\pi \xi t}dt\\ = & \;e^{j2\pi f_{p\_n}\left(kT_{sp}+\Delta \tau \right)}e^{j2\pi \xi (t_{mn}+kT_{sp}+\frac{\Delta \tau }{2})}e^{j\pi (f_{(m+k)n}+f_{mn})\Delta \tau }\\ \; & \;\left(T_{sp}-\Delta \tau \right)\mathrm{sinc}\left\{\left(T_{sp}-\Delta \tau \right)\left[f_{(m+k)n}-f_{mn}+\xi \right]\right\}.\; \end{array}$$

Analogously, the AF of the end part can be obtained.

(10) $$\begin{array}{cc} \; & \;\chi_{sp\_e}\left(m,n,k,\Delta \tau ,\xi \right)\\ = & \;\int_{\frac{T_{sp}}{2}+t_{mn}+kT_{sp}}^{\frac{T_{sp}}{2}+t_{mn}+\tau }e^{j2\pi f_{mn}\left(t-t_{mn}\right)}e^{j2\pi f_{p\_n}\left(t-t_{p\_n}\right)}e^{-j2\pi f_{mn}\left(t-t_{mn}-\tau \right)}e^{-j2\pi f_{p\_n}\left(t-t_{p\_n}-\tau \right)}e^{j2\pi \xi t}dt\\ = & \;e^{j2\pi f_{p\_n}\left(kT_{sp}+\Delta \tau \right)}e^{j2\pi \xi (t_{mn}+kT_{sp}+\frac{T_{sp}}{2}+\frac{\Delta \tau }{2})}e^{j\pi \left(f_{(m+k+1)n}+f_{mn}\right)\left(\Delta \tau -T_{sp}\right)}\\ \; & \;\Delta \tau \mathrm{sinc}\left\{\Delta \tau [f_{(m+k+1)n}-f_{mn}+\xi ]\right\}\; \end{array}$$

On the range profile at ξ=0, $\chi_{sp\_f}\left(m,n,k,\Delta \tau ,\xi \right)$ and $\chi_{sp\_d}\left(m,n,k,\Delta \tau ,\xi \right)$ contribute to the accumulation of the AF main lobe only when k=1 and k=−1, respectively. At this time, their expressions are unified as

(11) $$\begin{array}{cc} \chi_{sp\_f}\left(m,n,0,\tau ,0\right)\; & \;=\chi_{sp\_d}\left(m,n,-1,T_{sp}+\tau ,0\right)\\ \; & \;=\frac{\left(1-|\tau |B_{sp}\right)}{B_{sp}}\frac{\sin(\pi NMB_{sp}\tau )}{\sin(\pi B_{sp}\tau )}.\; \end{array}$$

The main lobe power is irrelevant to the frequency encoding of the DFC waveform. However, the side-lobe characteristics are closely related to the frequency encoding.

**Figure 4:**
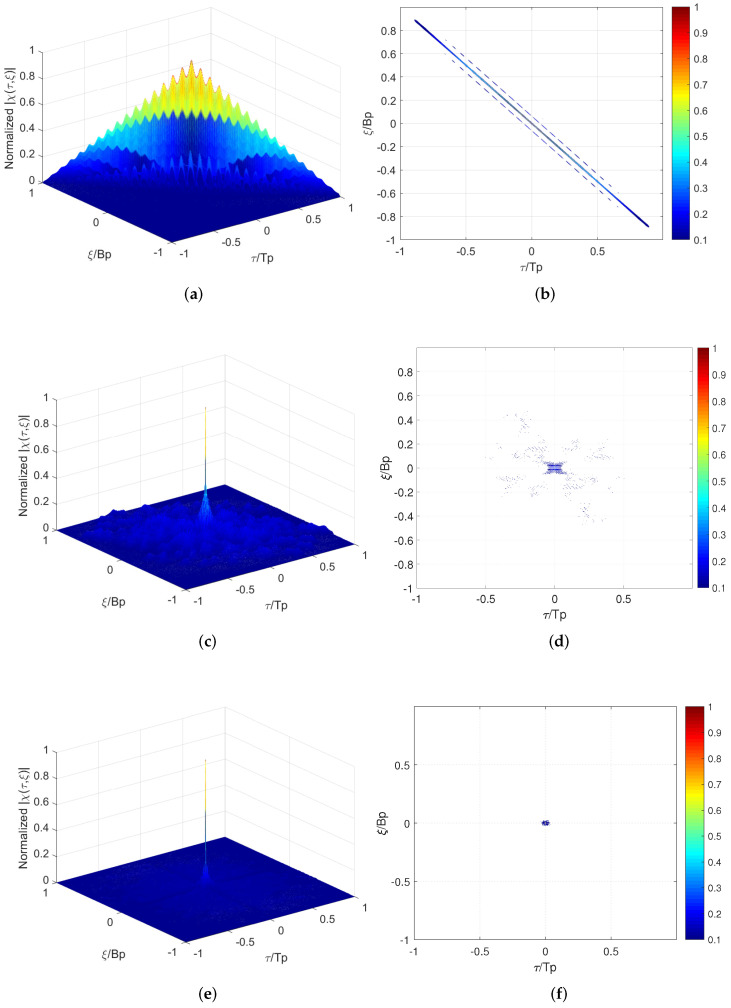
Ambiguity function plots(dB): (**a**) 3-D plot of the RSF-LS-DFC AF; (**b**) contour plot of the RSF-LS-DFC AF; (**c**) 3-D plot of the RSF-IC-DFC AF; (**d**) contour plot of the RSF-IC-DFC AF; (**e**) 3-D plot of the RSF-DC-DFC AF; (**f**) contour plot of the RSF-DC-DFC AF.

**Figure 5:**
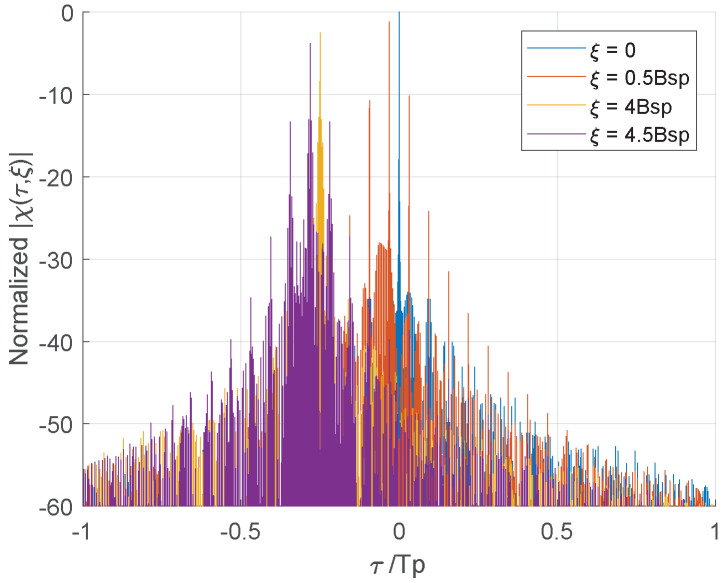
Comparison of different Doppler profiles in RSF-LS-DFC.

For a random stepped frequency modulated linear stepped-DFC waveform (RSF-LS-DFC), whose sub-pulses are stepped sequentially in each pulse, when the delay is an integer multiple of the sub-pulse width, that is $\tau =kT_{sp}$, AF only contains the summation of $\chi_{sp\_f}$. The multiple pairs of sub-pulses in the alignment part have a fixed frequency difference of $kB_{sp}$, which will lead to an accumulation of the AF side-lobes at $\xi =kB_{sp}$, forming the oblique ridge as shown in Figure 4a. For the non-integer time delays, the frequency coupling $\left(k+1\right)B_{sp}$ induced by the $\chi_{sp\_e}$ will increase the harmonics, which is reflected on both sides of the AF oblique ridge. This delay-Doppler coupling characteristic of RSF-LS-DFC is also reflected in the correlation function in Figure 5. On the Doppler profiles of integer multiples of $B_{sp}$, the correlation peak moves according to the range–Doppler coupling relationship, similar to the LFM waveform. On the non-integer $B_{sp}$ profile, as the harmonics on both sides of the peak are greatly elevated, the range focusing performance is significantly deteriorated.

For the random stepped frequency modulated identical Costas-DFC waveform (RSF-IC-DFC), each pulse adopts the same Costas array modulation. The frequency difference between the aligned Costas modulated sub-pulses are no longer fixed, which can eliminate the delay-Doppler coupling on the AF side-lobe. However, the identical Costas encoding induces side-lobe coherent accumulation between the multiple pulses during the frequency synthesis. So the side-lobe distribution will not be improved after the multi-pulses frequency synthesis, and it is still consistent with the single pulse performance.

**Figure 6:**
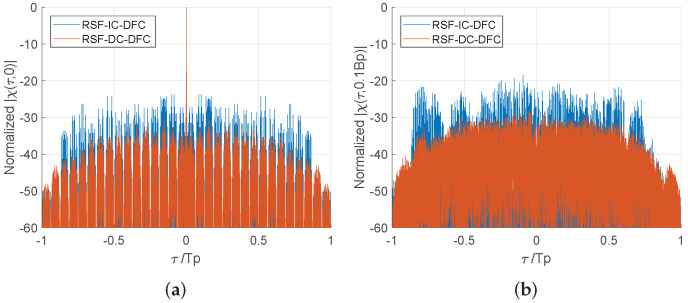
Comparison of RSF-IC-DFC and RSF-DC-DFC in different Doppler profiles: (**a**) 0-Doppler profile; (**b**) 0.1$B_{p}$-Doppler profile.

Similar to the RSF-IC-DFC, due to the use of intra-pulse Costas modulation, the AF of RSF-DC-DFC indicates no range–Doppler coupling but a approximate ideal thumbtack at the origin. Furthermore, the level of the side-lobe pedestal is significantly reduced by the application of different Costas arrays between pulses. Although the cross-correlation of these multiple Costas arrays are not optimal in this paper, but their different side-lobe distributions make it hard to form coherent accumulation after the frequency synthesis. Figure 6 shows the side-lobe performance improvement of RSF-DC-DFC on different Doppler profiles.

After the time width *T* and bandwidth *B* of the waveform are fixed, under the waveform division strategy, the selection of *N* determines the time width $T_{p}$ and $B_{p}$ of each pulse. According to the optimal criterion of the costas order *M*, $NM=\sqrt{TB}$ can be obtained. This means that the smaller *N* is, the larger *M* is. There are fewer waveform segments, and the duration and bandwidth of each pulse increases. We compared the sidelobe performance on the zero Doppler slice of the normalized AF plane with different values of *N* in Table 1. Two points need to be noted for the simulation related to *N*. First, since there is no 32-order Costas array, the 32-order sequence is generated by inserting the number 32 into a random position of the 31-order Costas array. Second, the total number of 4-order Costas arrays is only 12, so the pulses in the RSF-DC-DFC waveform randomly pick one of these 12 sequences, and there will be repetitions.

It can be seen from the results in Table 1 that the PLSR performance of the three waveforms is close. RSF-LS-DFC exhibits the best ISLR performance; however, the delay–Doppler coupling makes it susceptible to frequency shift interference. RSF-DC-DFC has better ISLR than RSF-IC-DFC. This is consistent with the results in the AF plot. The increase in ISLR indicates that the energy of the target is more widely dispersed in the imaging area, which will affect the imaging quality.

Hence, what we learn from the AF of RSF-DC-DFC is three-fold. Firstly the elimination of range–Doppler coupling can effectively resist the shift-frequency jamming. Secondly, lower side-lobe ensures the image quality when the amount of scattering becomes higher. Lastly, the sharp peak indicates poor Doppler tolerance, which will decrease the SAR imaging quality in the azimuth edge area.

## 3. SAR ECCM Based on RSF-DC-DFC

### 3.1. SAR ECCM Strategy

**Figure 7:**
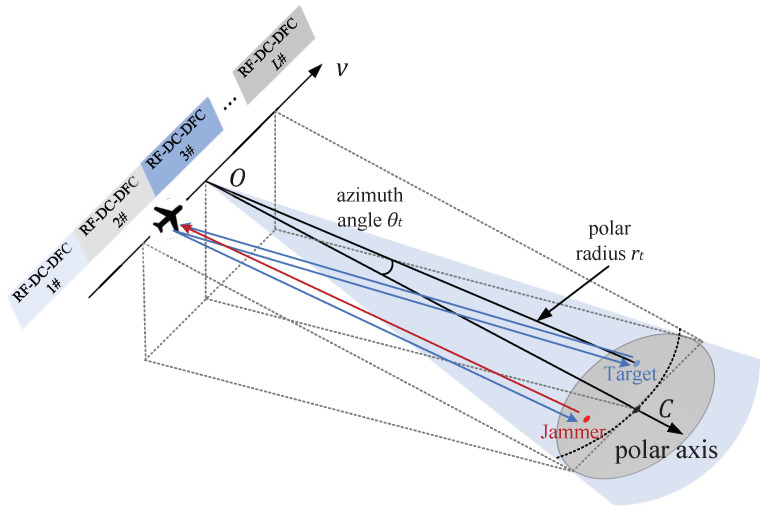
RSF-DC-DFC waveform application for SAR ECCM.

In this paper, a slant range plane is applied for SAR imaging, as shown in Figure 7. This is a side-looking imaging geometry. The solid line is the path of the antenna phase center (APC) which moves at a constant velocity of *v*. The imaging plane is the light blue slope formed by the APC path and the center of the gray imaging region *C*. On this slope, take the center *O* of the APC path as the pole, and take the line O−C as the polar axis; a polar coordinate system is established to represent the position of the target. The coordinates of point *C* are $\left[r_{c},0\right]$. The imaging result of the slant range plane can be projected onto the ground plane based on the geometric relationship according to actual needs.

The jammer located in the imaging region intercepts the radar signal and forwards it according to different deception jamming strategies. Both the jamming signal and the radar echo will be received by the airborne radar. Coherent jamming strategies such as deception and shift-frequency jamming will obtain a certain accumulation gain in two-dimensional imaging processing. The jammer can achieve two-dimensional multiple false targets or small-area mask by relatively low jamming power. However, due to the inherent processing delay of the jammer, it is safe to assume that the interfering signals are not superimposed on the echo of the current pulse. Considering that the RSF-DC-DFC waveform applied different frequency coding and random frequency modulation between pulses, SAR imaging based on this agile waveform is a very effective ECCM strategy. In this scenario, a total of *L* RSF-DC-DFC waveforms are sequentially transmitted during the APC motion.

The cost of waveform agility is the Doppler tolerance degradation. It will bring about a decrease in range-focusing performance when the radial velocity is unknown, especially at the azimuth edge of the imaging area. So, when RSF-DC-DFC is applied to counter the deception jamming of SAR, it is essential to specifically deal with the Doppler tolerance reduction in the SAR imaging processing.

### 3.2. Coherent SAR Imaging Processing

Next, we outline a coherent two-dimensional SAR imaging processing method that effectively improves the image quality degradation caused by the poor Doppler tolerance of RSF-DC-DFC. In this method, FCDC is integrated into a polar coordinate imaging algorithm before the frequency synthesis [29]. FCDC can solve the Doppler tolerance problem of RSF-DC-DFC without much extra computational complexity. The only constraint the FCDC imposes on the transmit waveform is that the corresponding pulses of the multiple RSF-DC-DFC within one measurement cycle need to be linearly correlated [30]. If we denote each pulse signal by a vector $p_{n}$, the RSF-DC-DFC waveform can be represented by a matrix. The expression for the *l*-th waveform in the measurement cycle is

(12) $$S_{l}=S\mathrm{diag}\left(\phi_{l}\right)=\left[\begin{array}{cccc} p_{1} & p_{2} & \cdots & p_{N} \end{array}\right]\left[\begin{array}{cccc} \phi_{1,l} & \cdots & 0 & 0\\ 0 & \phi_{2,l} & \cdots & 0\\ \vdots & \vdots & \ddots & \vdots \\ 0 & 0 & \cdots & \phi_{N,l} \end{array}\right],$$

where $\mathrm{diag}(\phi_{l})$ denotes the complex amplitudes diagonal matrix of the *l*-th RSF-DC-DFC waveform.

#### 3.2.1. Radius-Angle Decoupling in Frequency Domain

First, frequency-domain matched filtering is used to complete the deramp processing. The matched filter for each pulse is constructed based on the simulated echo of the imaging region center $S_{n,l}\left[t-\frac{2R_{c}\left(n,l\right)}{c}\right]$, where $R_{c}\left(n,l\right)$ is the polar radius history of *C* during the APC movement.

(13) $$R_{c}\left(n,l\right)=\sqrt{r_{c}^{2}+{\left\{\left[\left(n-1\right)+\left(l-1\right)N\right]T_{r}v\right\}}^{2}}$$

The expression of the target echo is $S_{n,l}\left[t-\frac{2R_{t}\left(n,l\right)}{c}\right]$. Similarly, $R_{t}\left(n,l\right)$ is the instantaneous range from the APC to the target $\left[r_{t},\theta_{t}\right]$.

(14) $$R_{t}\left(n,l\right)=\sqrt{r_{t}^{2}\cos^{2}\theta_{t}+{\left\{\left[\left(n-1\right)+\left(l-1\right)N\right]T_{r}v-r_{t}\sin\theta_{t}\right\}}^{2}}$$

A Fourier transform is performed on the received echo pulse and the corresponding match filter; the frequency domain product of $F[R_{t}\left(n,l\right)]$ and the conjugate of $F[R_{c}\left(n,l\right)]$ is the deramp signal without the residual video phase, which can be derived to be

(15) $$F_{deramp}\left(\omega ;n,l\right)={\left|f_{p\_n}\left(\omega \right)\right|}^{2}e^{-j\frac{2(\omega_{n}+\omega )}{c}\left[R_{t}\left(n,l\right)-R_{c}\left(n,l\right)\right]},$$

where $\omega_{n}=2\pi f_{p\_n}$.

$R_{t}\left(n,l\right)-R_{c}\left(n,l\right)$ is expanded along the slow time at the center of the APC path in this section, and its first-order Taylor approximation contains the coarse Doppler information. Then, the equation above can be written as

(16) $$F_{deramp}\left(\omega ;n,l\right)\approx {\left|f_{p\_n}\left(\omega \right)\right|}^{2}e^{-j\frac{2(\omega_{n}+\omega )}{c}\left\{r_{t}-r_{c}-v\sin\theta_{t}\left[\left(n-1\right)+\left(l-1\right)N\right]T_{r}\right\}}.$$

A keystone resampling is applied here to calibrate the radius migration by slow-time interpolation as shown in (17). It eliminates the interSection of ω and θ, realizing the radius–angle decoupling successfully.

(17) $$t_{n,l}=\frac{\left(\omega_{n}+\omega \right)\left[\left(n-1\right)+\left(l-1\right)N\right]T_{r}}{\omega_{c}},$$

where $\omega_{c}=2\pi f_{c}$.

The decoupled signal can be expressed as follows:

(18) $$F_{ra}\left(\omega ;n,l\right)={\left|f_{p\_n}\left(\omega \right)\right|}^{2}e^{-j\frac{2}{c}\left[\left(\omega_{n}+\omega \right)\left(r_{t}-r_{c}\right)-\omega_{c}v\sin\theta_{t}t_{n,l}\right]}.$$

In (18), the angle-dependent phases associated with the coarse Doppler varies with pulse time, which results in phase discontinuity when frequency synthesis is performed on the multiple pulses modulated by random frequencies. Considering the rank 1 constraint of multiple RSF-DC-DFC waveforms, the Doppler information between these waveforms can be extracted to correct the angle-dependent phases between multiple pulses within each RSF-DC-DFC waveform.

#### 3.2.2. Radius Focus Based on FCDC

The polar coordinate transformation keeps the Doppler characteristic consistency within and between the multiple RF-DC-DF waveforms. Based on the consistency mentioned above, the Doppler information between these multiple waveforms can be extracted to correct the Doppler modulation within each RSF-DC-DFC waveform.

FCDC is designed to be performed on a discrete 2D grid. The *q*-th sampling point of each decoupled signal $F_{ra}\left(q\Delta \omega ;n,l\right)$ is extracted to form Q two-dimensional matrices of size N×L.

A discrete Fourier transform (DFT) over the waveform slow time will compress each target into its corresponding Coarse Doppler column. The position of the coarse Doppler train corresponds to the phase relationship between the multiple pulses within the waveform.

The angle-dependent phase introduced by the coarse Doppler on all grids can be corrected by a element-wise multiplication with matrix C.

(19) $$C=e^{-j2\pi \frac{\left(n-1)(l-1\right)}{NL}}\in C^{N\times L}$$

After all the Q matrices complete the FCDC processing, the signal spectrum is rearranged and concatenated according to the pulse modulation frequency on each Doppler column. The signal expression on the target Doppler column is

(20) $$F_{rd}\left(\omega \right)\approx \sum_{n=1}^{N}\mathrm{rect}\left[\frac{\omega -\left(n-1\right)\omega_{p}}{\omega_{p}}\right]{\left|{\hat{F}}_{n}\left(\omega \right)\right|}^{2}e^{-j\frac{2}{c}\left({\hat{\omega }}_{n}+\omega \right)\left(r_{t}-r_{c}\right)},$$

where $\omega_{p}=2\pi B_{p}$, ${\hat{\omega }}_{n}$ and ${\hat{F}}_{n}\left(\omega \right)$ are the modulation frequency and the corresponding frequency domain of the rearranged pulse, respectively.

The radius compression can be obtained by an inverse Fourier operation in the frequency domain. The focused signal can be calculated as

(21) $$S_{r}\left(t\right)\approx \chi \left[t-\frac{2(r_{t}-r_{c})}{c},0\right]e^{-j\omega_{c}\frac{2(r_{t}-r_{c})}{c}}.$$

#### 3.2.3. Angle Focus

In order to achieve precise focusing on the polar angle dimension, we need to restore the slow time information of the radius-compressed signal through inverse Fourier operation. The recovered signal contains the historical range differences based on the waveform slow time as below.

(22) $$S_{rt}\left(t,l\right)\approx \chi \left[t-\frac{2(r_{t}-r_{c})}{c},0\right]e^{-j\frac{2w_{c}}{c}\left[R_{t}\left(\frac{N}{2},l\right)-R_{c}\left(\frac{N}{2},l\right)\right]}$$

According to the conclusion of [31], when the range differences above is expanded based on the polar angle, it is sufficient to use Taylor’s first-order approximation in the imaging scene with small azimuth angle.

(23) $$S_{rt}\left(t,l\right)\approx \chi \left[t-\frac{2(r_{t}-r_{c})}{c},0\right]e^{-j\frac{2w_{c}}{c}\left\{{\hat{R}}_{t}\left(\frac{N}{2},l\right)-R_{c}\left(\frac{N}{2},l\right)-\frac{r_{t}v\left[\frac{N}{2}+\left(l-1\right)N\right]T_{r}}{{\hat{R}}_{t}\left(\frac{N}{2},l\right)}\theta_{t}\right\}}$$

where ${\hat{R}}_{t}\left(\frac{N}{2},l\right)$ refers to the historical slope range of $\left[r_{t},0\right]$ at the central time of each RSF-DC-DFC waveform. Radius focusing enables the phase compensation and the angle interpolation according to radius gates $r_{t}$. The phase compensation and the angle interpolation formulas are as follows:

(24) $$S_{pc}\left(t,l\right)=\delta \left[t-\frac{2(r_{t}-r_{c})}{c}\right]e^{j\frac{2w_{c}}{c}\left[{\hat{R}}_{t}\left(\frac{N}{2},l\right)-R_{c}\left(\frac{N}{2},l\right)\right]},$$

where δ(t) is the impulse function.

(25) $$t_{l}=\frac{r_{t}\left[\frac{N}{2}+\left(l-1\right)N\right]T_{r}}{{\hat{R}}_{t}\left(\frac{N}{2},l\right)}$$

Finally, the Fourier transform is applied to complete the angle compression, and the focused target can be obtained in the polar coordinate system of the imaging slope.

(26) $$S\left(r,\theta \right)=\chi \left\{\frac{2}{c}\left[r-\left(r_{t}-r_{c}\right)\right],0\right\}\times \mathrm{sinc}\left[\frac{2vf_{c}}{c}\left(\theta -\theta_{t}\right)\right]$$

**Figure 8:**
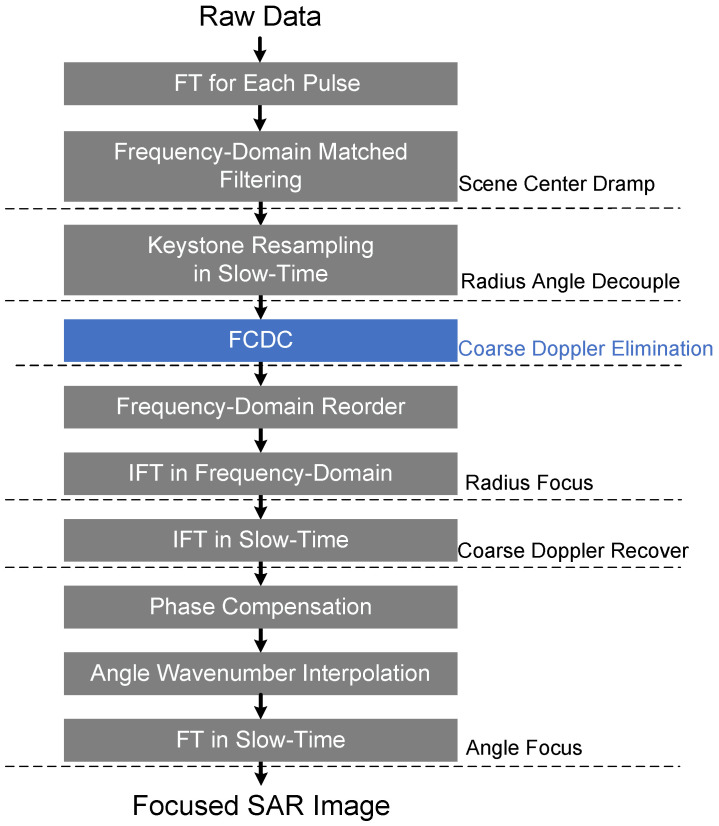
Flowchart of the coherent imaging processing.

The complete coherent SAR imaging flowchart based on RSF-DC-DFC waveform are shown in Figure 8.

## 4. Simulation Results

For performance analysis of RSF-DC-DFC waveform SAR, Ku-band parameters are employed to execute the simulations, as listed in Table 2.

### 4.1. FCDC Algorithm Performance Verification

**Figure 9:**
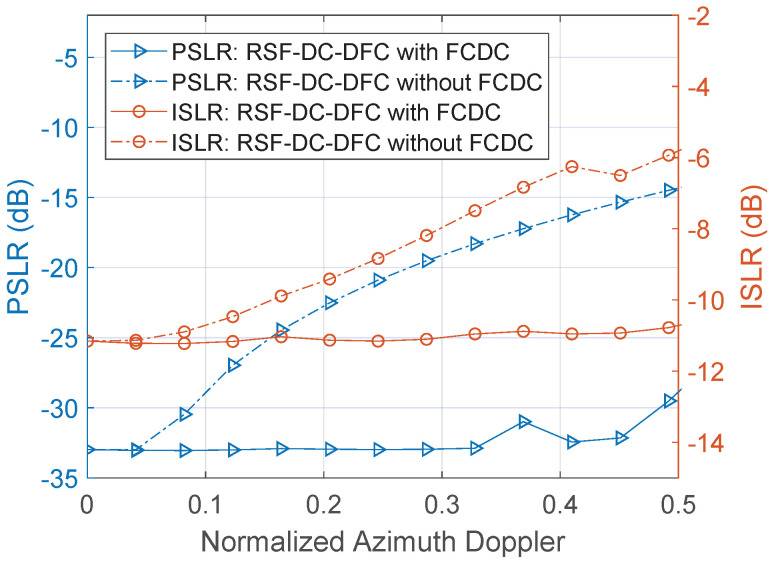
PSLR and ISLR as a function of the normalized Coarse Doppler.

First, simulations are performed in a noiseless setup to observe the improvement effect of the algorithm embedding FCDC on the RSF-DC-DFC SAR imaging. We start from analysis of the radius profile for a target with different normalized coarse Doppler from 0 to 0.5 and a constant Center slant range. The normalized azimuth Doppler here corresponds to the azimuth angle with this configuration. Considering that the azimuth Doppler mainly affects the frequency synthesis performance, Figure 9 shows the variation of PSLR and ISLR on the radius profile when the normalized azimuth Doppler changes.

Clearly, due to the Doppler sensitivity of the RSF-DC-DFC waveform, when the target azimuth increases, the radius focusing performance decreases due to the Doppler introduced by the high-speed motion of the platform. It leads to serious defocusing for the targets on the azimuth edge of the scene. Adding FCDC to the algorithm can effectively improve the effect of the azimuth Doppler on focusing performance. When the normalized Azimuth Doppler increases to 0.4, the radial PSLR and ISLR are improved by 16.2 dB and 4.7 dB, respectively, after using the FCDC algorithm.

The Doppler FFT in FCDC successfully handles the poor Doppler tolerance problem by coherent processing gain. The introduction of FCDC eliminates the radius focusing deterioration with the increasing azimuth angle, and the radius focusing performances in different azimuths are basically consistent. Evidently, since FCDC is executed under discrete Doppler cells, there will be some Doppler residues. However, in general, when *L* is greater than 10, these residues are negligibly small, and can be ignored in the practical applications.

### 4.2. Image Performance with Different N and M

The AF represents the ideal correlation characteristics of the waveform. However, the approximation processing and the out-of-band leakage of each pulse during the frequency synthesis will change the image sidelobe characteristics. In this section, the simulation is set to observe the sidelobe performance of the point target radius slice in different waveform images.

As can be seen from the results in Table 3, the overall performance of RSF-DC-DFC is still better than that of RSF-IC-DFC. At the same time, it can be found that, the smaller *N* is, the better the sidelobe performance of the waveform is.

When N becomes smaller, the number of waveform segments decreases and the pulse bandwidth increases accordingly. At this time, the frequency hopping distribution of the overall waveform is more flexible, resulting in better sidelobe performance. However, the problem brought by the decrease in *N* is that the pulse energy enhancement will raise the risk of interception by the jammer. ISRJ is more likely to occur within the echo admission window of the current pulse, and is difficult to overcome for the waveform itself. Moreover, the wider pulse bandwidth lifts the baseband signal processing requirement. On the contrary, bigger *N* will decrease the image side lobe performance. However, it will enhance the anti-interference performance of the waveform and have higher computational efficiency. Therefore, the selection of *N* needs a trade-off between the imaging performance, the anti-jamming capability and the baseband hardware requirements.

### 4.3. Anti-Jamming Performance Simulation

In this section, simulations based on different jamming strategies are designed to verify the anti-jamming performance of RSF-DC-DFC SAR.

#### 4.3.1. ISRJ ECCM Simulation

Frequency Shift Keying Costas Coding (FSK-Costas) [20] was adopted as a comparison waveform, which has 102.4 μs duration and 640 MHz bandwidth. Compared with the RSF-DC-DFC, the parameter setting allows these two waveforms to have the same coherence gain, range resolution and azimuth resolution. ISRJ jammer intercepts the transmitted signal for 10 μs duration and retransmits it at a 50% duty cycle. The out-of-band rejection of the baseband filter in RSF-DC-DFC is greater than 18 dB.

**Figure 10:**
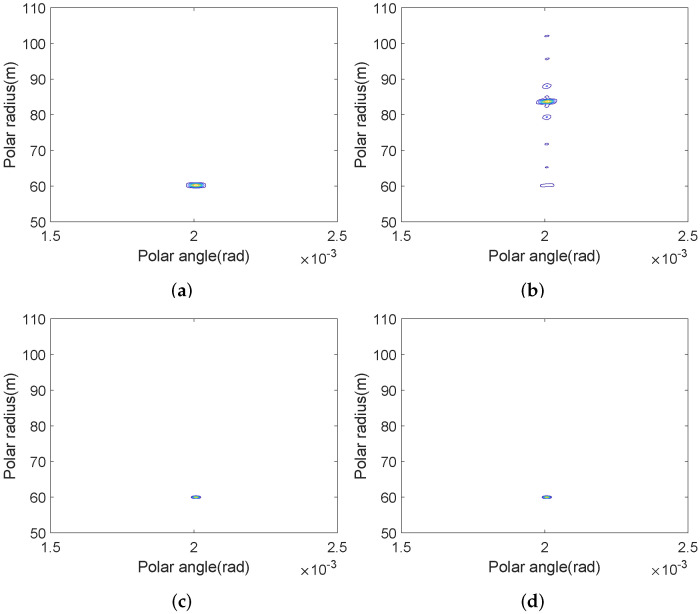
Image scene composed of 1 point at [60 m, 0.002 rad](dB): (**a**) FSK-Costas SAR without ISRJ; (**b**) FSK-Costas SAR with ISRJ; (**c**) RSF-DC-DFC SAR without ISRJ; (**d**) RSF-DC-DFC SAR with ISRJ.

**Figure 11:**
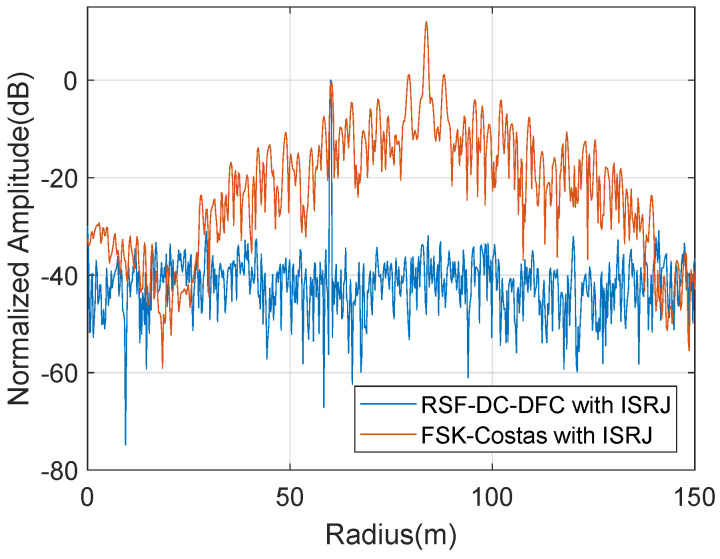
Radius slice comparison.

It can be seen from Figure 10b that, since the ISRJ interference can act on the current pulse of FSK-Costas, the wideband pulse is sampled and forwarded at subsection intervals. Hence the interference with strong coherence can form multi-point interference in the radius dimension. The maximum interference is about 12 dB higher than the true target. ISRJ can induce similar jamming effects on the other wideband waveforms. While RSF-DC-DFC can utilize the time segment and frequency diversity between the pulses to combat ISRJ. From the results in Figure 10d, it can be seen that the interference has little effect on the RSF-DC-DFC SAR image. Figure 11 is the radius slice comparison of Figure 10a,b.

#### 4.3.2. Frequency Shift Jamming ECCM Simulation

ECCM simulations against frequency shift jamming are designed with RSF-LS-DFC, RSF-IC-DFC and RSF-DC-DFC separately. There is no phase modulation among the multiple RSF-LS-DFC waveforms, considering that these three waveforms decompose the traditional wideband signal into N sub-band pulses to be sent at intervals.

It is difficult for the jamming signal to act on the current pulse for the relatively short pulse width. Due to the random frequency agility between the pulses in each waveform, it will lead to invalid interference based on the direct or repeated forwarding of the intercepted pulses. It should be noticed that the frequency hopping patterns of the multiple consecutive waveforms are consistent. So, if the jammer can identify the waveform cycle containing multiple pulses, and all the pulses in the entire waveform are intercepted and forwarded to the next waveform cycle, it is possible to form effective interference.

Therefore, it is assumed that the duty cycle of the SAR waveform can be accurately obtained by the jammer in the following simulation. The jammer can intercept all the pulses of the waveform, and perform retransmission based on different frequency shift jamming strategies during the subsequent waveform period. At the same time, the jammer signal maintains coherence during the operation of the multiple waveforms. There are three types of jamming strategies: fixed frequency shift interference (FFSI), random frequency shift interference (RFSI) and stepped frequency shift interference (SFSI).

**Figure 12:**
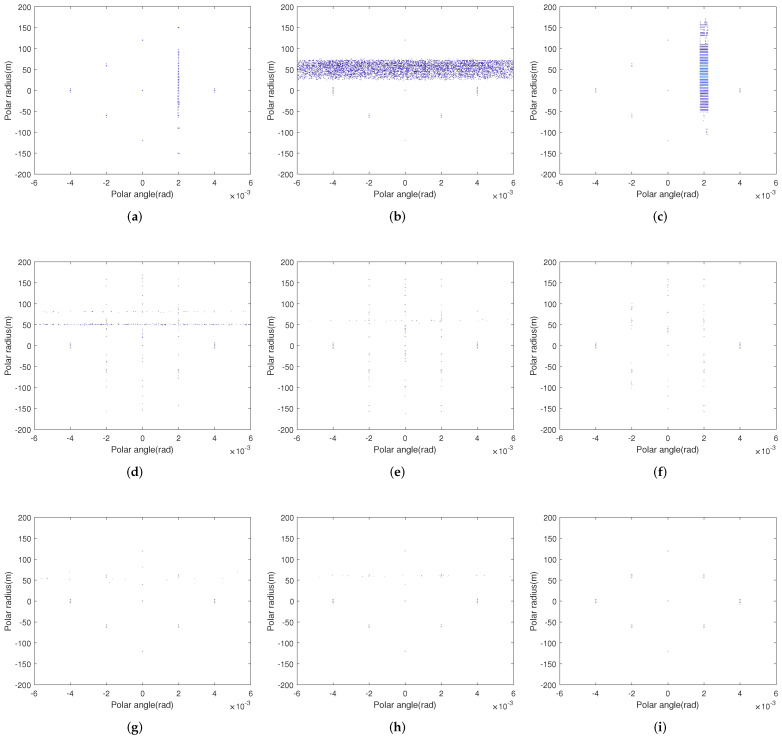
Image scene composed of 9 points: (**a**) RSF-LS-DFC SAR with FFSI; (**b**) RSF-LS-DFC SAR with RFSI; (**c**) RSF-LS-DFC SAR with SFSI; (**d**) RSF-IC-DFC SAR with FFSI; (**e**) RSF-IC-DFC SAR with RFSI; (**f**) RSF-IC-DFC SAR with SFSI; (**g**) RSF-DC-DFC SAR with FFSI; (**h**) RSF-DC-DFC SAR with RFSI; (**i**) RSF-DC-DFC SAR with SFSI.

As shown in Figure 12, since RSF-LS-DFC has the characteristic of delay–Doppler coupling, and there is no phase modulation among its multiple waveforms, the jamming peaks and larger harmonics in the radius profile constitute the pattern of multi-point interference under FFSI. Multi-point interference is distributed near the radius of the target to be protected. Under the remaining two interference strategies, strip and mask jamming areas appear in the imaging region, respectively. Both barrage effects successfully achieve the occlusion of the target to be protected. Due to the costas-DFC waveform structure in pulse, there is no frequency delay coupling in RSF-IC-DFC and RSF-DC-DFC. Therefore, the energy of the frequency-shifted interference cannot be accumulated during the frequency-domain matched filtering process. At the same time, the phase modulation between the multi-waveform pulses also makes it difficult for the jamming signal to accumulate in the azimuth. That is to say, these two waveforms can effectively resist frequency-shift interference. However, due to the higher sidelobe of RSF-IC-DFC, the imaging quality is significantly worse than that of RSF-DC-DFC.

**Figure 13:**
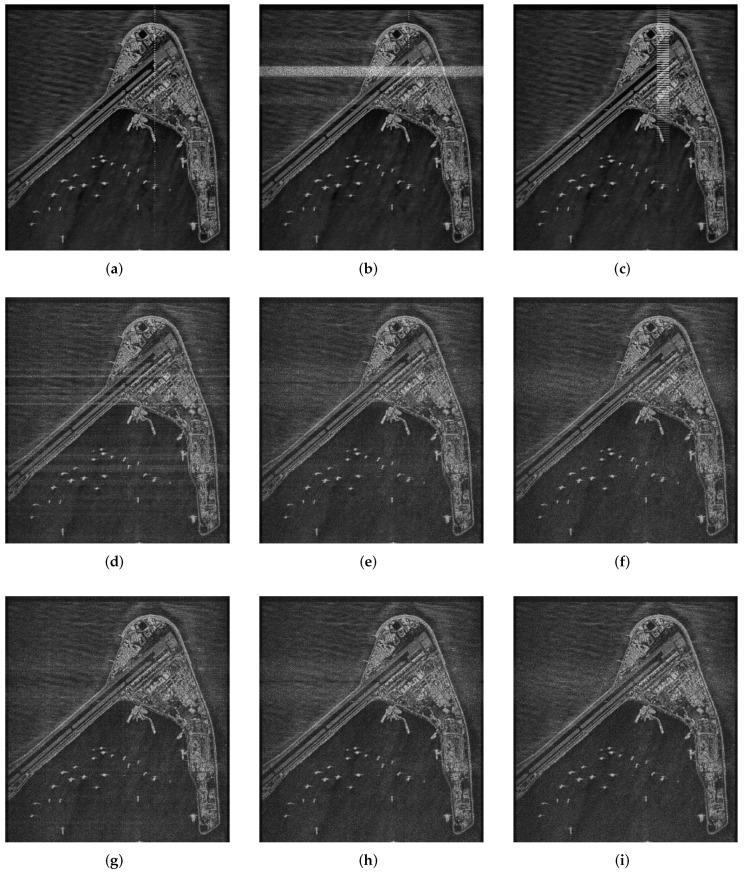
Simulation images of a real scene: (**a**) RSF-LS-DFC SAR with FFSI; (**b**) RSF-LS-DFC SAR with RFSI; (**c**) RSF-LS-DFC SAR with SFSI; (**d**) RSF-IC-DFC SAR with FFSI; (**e**) RSF-IC-DFC SAR with RFSI; (**f**) RSF-IC-DFC SAR with SFSI; (**g**) RSF-DC-DFC SAR with FFSI; (**h**) RSF-DC-DFC SAR with RFSI; (**i**) RSF-DC-DFC SAR with SFSI.

Finally, SAR raw data simulations of Subi Reef scene were carried out in Figure 13 to verify the anti-interference ability of the proposed RSF-DC-DFC waveform. Apparently, RSF-DC-DFC waveform benefits from the agile characteristics of its pulses in the frequency coding, carrier frequency and phase compared with the other two waveforms. Even if the pulse period and frequency hopping pattern are estimated by the jammer accurately, the jamming signal can hardly form a focus false target or regional mask in the SAR images when RSF-DC-DFC waveform is employed. RSF-IC-DFC waveform shows a similar anti-interference effect in SAR imaging, but due to its poor sidelobe characteristics, the imaging quality is worse than that of RSF-DC-DFC waveform. In addition, inserting FCDC into the signal processing ensures that details and contours can be obtained at the edge of the imaging area.

The structural SIMilarity (SSIM) [32] is applied here to evaluate the image quality. The SSIM comparisons for RSF-IC-DFC, RSF-DC-DFC and different jamming strategies are listed in Table 4, where a larger SSIM value implies a better image quality. Both of these waveforms will not produce obvious point-like or stripe-like interference patterns facing the frequency shift interference. The original scene of LFM without jamming is chosen as the benchmark. From the table, we can verify the validity of FCDC. It also shows that RSF-DC-DFC has better imaging capabilities.

## 5. Conclusions

A multi-pulse wideband waveform RSF-DC-DFC is proposed based on waveform diversity in this paper. This waveform divides the wideband waveform into multiple narrow band pulses; each pulse has an agile carrier frequency, flexible frequency coding and phase modulation. The jamming signals can barely accumulate between those multiple pulses. So, the RSF-DC-DFC waveform can be successfully applied in the interference countermeasures of SAR imaging. Moreover, considering the Doppler-sensitive characteristics of this waveform, a special FCDC is inserted in the signal processing flow to realize the coherent frequency synthesis after the full-scene Doppler correction, which ensures the imaging quality of the edge area. Simulations based on different jamming strategies and imaging scenarios are performed. The effectiveness of this waveform and corresponding signal processing algorithm against coherent jamming have been validated.

## Figures and Tables

**Table 1:** Comparison of the AF sidelobe performance with different N and M.

*N*	*M*	RSF-LS-DFC		RSF-IC-DFC		RSF-DC-DFC	
		PSLR (dB)	ISLR (dB)	PSLR (dB)	ISLR (dB)	PSLR (dB)	ISLR (dB)
2	128	−13.2626	−9.6820	−13.3170	−3.3939	−13.3249	−3.7662
4	64	−13.2598	−9.6865	−13.3560	−3.4875	−13.4200	−4.1352
8	32	−13.2509	−9.7043	−13.5070	−3.8768	−13.5050	−4.4049
16	16	−13.2319	−9.7717	−14.0080	−3.8718	−13.9331	−4.7979
32	8	−13.2565	−10.0172	−14.5996	−5.2644	−14.4600	−5.3495
64	4	−13.9637	−10.8429	−13.4648	−5.6608	−14.3857	−6.5389

**Table 2:** Simulation parameter.

Parameter	Value
Carrier frequency ($f_{c}$)	16 GHz
Pulse width ($T_{p}$)	6.4 μs
Costas array order (*M*)	16
Pulse number in each RSF-DC-DFC (*N*)	16
RSF-DC-DFC waveform number (*L*)	1536
Pulse bandwidth ($B_{p}$)	40 MHz
Pulse repetition frequency ($1/T_{r}$)	28 kHz
Center slant range	30,000 m
Platform velocity	1000 m/s
Signal-to-jammer ratio (SJR)	−18 dB
Target location to be protected	(60 m, 0.002 rad)

**Table 3:** Comparison of the image sidelobe performance with different *N* and *M*.

*N*	*M*	RSF-IC-DFC		RSF-DC-DFC	
		PLSR (dB)	ISLR (dB)	PLSR (dB)	ISLR (dB)
2	128	−43.1562	−17.4813	−43.7842	−17.6390
4	64	−37.0908	−15.2512	−40.6128	−15.3138
8	32	−29.7246	−12.8691	−32.5786	−13.0405
16	16	−25.6970	−10.2922	−34.741	−11.3919
32	8	−17.7007	−8.4286	−31.1683	−9.4307
64	4	−11.7092	−7.8878	−18.1193	−7.7786

**Table 4:** Comparison of the image quality in SSIM.

Waveform	Without FCDC	With FCDC			
	No Jamming	No Jamming	FFSI	RFSI	SFSI
RSF-IC-DFC	0.8896	0.9011	0.8588	0.8555	0.8566
RSF-DC-DFC	0.8947	0.9064	0.8624	0.8598	0.8610
